# Mechanical Dentistry at the Meeting of the American Association

**Published:** 1877-09

**Authors:** L. P. Haskell


### MECHANICAL DENTISTRY AT THE MEETING OF THE AMERICAN ASSOCIATION.

BY L. P. HASKELL.

A regular attendant upon the recent Annual Meeting of the American Dental Association, I was greatly interested in many of the papers presented, and in discussions which followed. Although not engaged in operative dentistry, I could fully appreciate the able presentations of the various subjects, showing, as many of them did, close study and research ; and then coming, as some of the ablest of them did, from the younger members of the profession. It showed that the places sooner or later to be vacated by the older members, will be filled, and so research and investigation in Dental science will keep pace with that of the cognate sciences.

In common, however, with many others, after taking especial pains to be present when the subject of Mechanical Dentistry was reached, I was greatly disappointed to find that it was entirely ignored, and passed with just a few lines from the chairman of the committee, as a postscript to a letter upon another subject, and not a word from the other members of the same committee.

Well, I thought, after a little reflection, this is a complete reflex of the position of the profession generally upon this subject, and no wonder that mechanical dentistry is degraded to so low a position, and left to the tender mercies of the "$8" tooth carpenters, now preying upon the public.

The cause of this unfortunate condition of things is very apparent to any one who gives the subject any consideration.

Twenty years ago, when only metal plates were used, it required skill to make a set or partial set of teeth. The introduction of vulcanized rubber has rendered that no longer necessary. It is now a simple mechanical operation, for one having but little knowledge of dentistry can put together the ready-made teeth and a piece of rubber. The use of moulded "gum sections" makes it impossible to display artistic taste in the arrangement of teeth, even though the aforesaid "tooth carpenters" had any, and they are as guiltless of it as they are of any correct knowledge of dentistry in general.

The very common representation among the profession that rubber is at least as good as anything for artificial dentures, has made it necessary for them to compete with these Ishmaelites, and as a result the best men in the profession, who have a good operative practice will not do it, and so allow that branch of their practice to go by default, except where they can get a remunerative price from some one who appreciates good work, so that they can use such methods and materials as will accomplish desired results.

The great mass of dental students, since the introduction of rubber, have had very limited instruction and practice in metal work, and as much practice is necessary to make it a success, it has fallen into very general disrepute among the new practitioners.

The profession should take hold of this subject in earnest, and educate their patients to see that it is impossible to secure such results without devoting time, skill, and other materials and methods, than rubber and celluloid.

In another article I will call attention to the mischievous effects of rubber in producing constant absorption of the alveolus.

I am glad to see that Dr. H. H. Keith, of St. Louis, one of the best mechanical dentists in the country, has been appointed chairman of the Committee on Mechanical Dentistry; and you may be sure that the next meeting of the Association will have a report upon this subject that will invite discussion and awaken new interest.

